# Symmetry-Breaking Drop Bouncing on Superhydrophobic Surfaces with Continuously Changing Curvatures

**DOI:** 10.3390/polym13172940

**Published:** 2021-08-31

**Authors:** WooSeok Choi, Sungchan Yun

**Affiliations:** Department of Mechanical Engineering, Korea National University of Transportation, Chungju 27469, Korea; w.choi@ut.ac.kr

**Keywords:** superhydrophobic surface, anisotropic surface, interfacial dynamics

## Abstract

Controlling the residence time of drops on the solid surface is related to a wide spectrum of engineering applications, such as self-cleaning and anti-icing. The symmetry-breaking dynamics induced by the initial drop shape can promote drop bouncing. Here, we study the bouncing features of spherical and ellipsoidal drops on elliptical surfaces that continuously change curvatures inspired by natural succulent leaves. The bounce characteristics highly depend on the geometric relations between the ellipsoidal drops and curved surfaces. Numerical results show that ellipsoidal shapes of the drops amplify asymmetries of the mass and momentum in synergy with an influence of the surface curvature during the impact, which is verified by experiments. Effects of the surface anisotropy and drops’ ellipticity on the residence time are investigated under various surface morphologies and Weber numbers. The residence time is closely associated with an initial surface curvature at the apex. The underlying principle of modifying the residence time via the drops’ ellipticity and initial surface curvature is elucidated based on momentum asymmetry. The understanding of the bouncing features on curved surfaces will offer practical implications for enhanced heat transfer performances and controlled water repellency, etc.

## 1. Introduction

Bouncing dynamics of drops on solid surfaces have gained substantial attention over the last two decades for industrial applications, such as self-cleaning [1], anti-icing [2], low friction [3], and dropwise condensation [4]. The bounce characteristics are highly dependent on the surface roughness, temperature, wettability, and ambient conditions [5,6]. The residence time is considered essential because it determines the extent to which mass, momentum, and energy are exchanged between surfaces and drops. Drops impacting on superhydrophobic surfaces can lift off quickly because of the low wetting hysteresis at the contact line, inspired by effects of the lotus leaves and pitcher plant [7,8]. The residence time can be shortened to the inertio-capillary time scale of (*ρD*^3^/*σ*)^1/2^ with circular symmetry, where *ρ*, *D*, and *σ* are the density of liquid, diameter of drop, and interfacial tension, respectively [9,10,11]. The emphases of recent works focus mainly on compelling drops to depart from the surfaces as fast as possible.

Many efforts have been devoted to further reducing the residence time by utilizing surface textures and chemical compositions. The surface morphologies were relevant to the sub-millimeter textures decorated on flat substrates, including center-assist bouncing along macro-ridge structures [12,13] and complicated cross-shaped macro-textures [14], counter-intuitive bouncing on lattice patterns of posts with nanostructures [15], and water-ring bouncing on point-like defects [16]. The research progress in this field developed some fresh regimes to enhance drop mobility based on the surface modifications.

Along with the development, recent works have demonstrated asymmetric bouncing dynamics on surfaces with the radius of curvature comparable to drop size, including asymmetric bouncing on tubular surfaces and natural succulent leaves [17], ribbed-curved surfaces [18], and cylindrical ridges in millimetric size [19,20]. The shape and size of the cylindrical ridges altered the drops’ behavior significantly, and hence the residence time could reduce further, compared with the dynamics on the flat surfaces. Particularly, there are two distinct regimes where the variation of the ridge in size has reverse effects on the residence time [19]. For cylindrical ridges greater than the drop size [17], the mass and momentum redistribute in the manner of a hydrodynamic interplay between the momenta in the tangential direction along the curve side and axial direction. The lift-off time further reduced at higher curvatures because the bouncing dynamics were closely related to the anisotropic curvature of the surface. On the contrary, when the ridge is much smaller than the drop size [12,13], a role of the flat substrate on which the ridge laid would be played in an interaction with the drops. After the impact, the drop split into two parts that retracted and bounced separately. Further demonstrations on Y-shaped or cross-shaped macro-texture surfaces reported that drops could be configured into several subunits and take off at the reduced residence time [13,14]. The lift-off time decreased as the ridge size increased in this regime [19].

Another methodology for the control of the residence time with non-spherical shaping has been suggested by our group [21]. The symmetry-breaking dynamics induced by the initial drop shape could potentially open up opportunities for modifying the impact dynamics. Ellipsoidal shapes allowed the peculiar spreading and retraction behaviors, thereby forming liquid alignment with the principal axis during retraction [21]. When the ellipsoidal drops were colliding on the heated [22] and superhydrophobic surfaces [23], the drops produced the preferential flow to the minor axis of the initial elliptical footprint, which led to the decrease in the residence time and bounce height. The distinguishable features of shape-dependent dynamics could significantly change the outcome of impact without target surface modification or additional chemical composition of the liquid. 

However, most of the previous works focused their attention solely on the scenarios of drop impact on flat substrates, and the effect of the initial drop shapes on hydrodynamics on anisotropic surfaces has yet to be explored [21,22,23]. Furthermore, there was a lack of knowledge on how shape distortions of impinging drops had an influence on the bouncing characteristics on the surfaces in practical spraying systems. Initial drop shapes would potentially amplify asymmetries of the mass and momentum in synergy with an influence of the surface curvature, which can alter the residence time. The recent study of our group investigated the bouncing features of the ellipsoidal drops on superhydrophobic cylinders and reported a further decrease in the residence time, compared with spherical drops impacting on the surfaces [24]. However, the latter study showed the shape-dependent dynamics on curved surfaces that were limited to circular cylinders. The knowledge may still be insufficient for practical applications, such as self-cleaning and biomimetic strategies.

**Figure 1:**
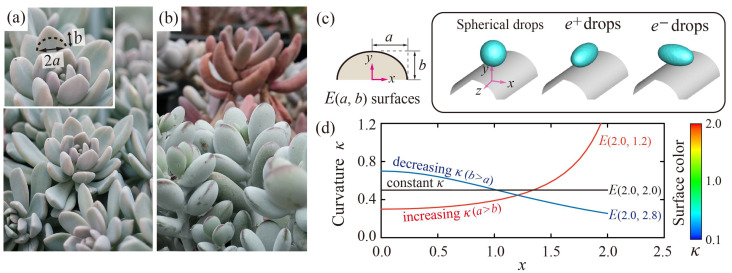
Surface morphologies of the natural succulent leaves and elliptical models. (**a**) *Echeveria*, (**b**) *Pachyphytum* (top), and *Cotyledon* (bottom) leaves showing the continuously changing radii of curvature in the millimetric scale. (**c**) Schematics of the elliptically curved surfaces (*E*-surfaces) and three geometric relationships between the surfaces and drops. (**d**) Dimensionless surface curvatures, *κ*, along the *x* axis. The color bar on the right indicates the relative magnitude of the curvature.

The current work was motivated by the symmetry-breaking bouncing on the *Echeveria* succulent leaves [17], which can be represented as the bouncing on elliptically curved surfaces in a more realistic situation, as captured in Figure 1a. The other leaves we found also exhibited the curved surfaces that change curvature along the surfaces, as shown in Figure 1b. To understand how the initial drop shape and surface curvature affect the dynamics, we study the bouncing features of the drops on elliptically curved surfaces (*E*-surfaces). The current work focuses on the above-mentioned hydrodynamic regime where the drops never contact the flat surface on which the ridge laid, and the ridge width is equal to or more than two-fold of the drop size. The influences of the drops’ ellipticity (*e*) and surface curvature (*κ*) on the residence time are scrutinized under various surface curvatures and impact velocities. In the momentum analysis, we discuss how the momentum asymmetry is relevant to the decrease in the residence time.

## 2. Materials and Methods

In the simulation, the volume of fluid (VOF) methods were employed to study the bouncing dynamics affected by the surface curvature and drops’ ellipticity. Water and air at room temperature and atmospheric pressure were chosen as operating fluids. The fraction of volume was represented as *ψ*. Overall, schemes were based on our earlier work and literatures that predicted the drop impingement on a substrate [21,25,26]. The unsteady and incompressible mass and momentum equations were coupled in the computational domain as:

(1) $$\frac{\partial }{\partial t}\left(\rho \right)+\nabla \cdot \left(\rho \vec{v}\right)=0$$

(2) $$\frac{\partial }{\partial t}\left(\rho \vec{v}\right)+\nabla \cdot \left(\rho \vec{v}\vec{v}\right)=-\nabla p+\nabla \cdot \left[\mu \left(\nabla \vec{v}+{\left(\nabla \vec{v}\right)}^{T}\right)\right]+\rho \vec{g}+2\sigma \rho \gamma \nabla \psi_{2}/\left(\rho_{1}+\rho_{2}\right)$$

where $\rho =\psi_{2}\rho_{2}+\left(1-\psi_{2}\right)\rho_{1}$, $\mu =\psi_{2}\mu_{2}+\left(1-\psi_{2}\right)\mu_{1}$, and *γ* = −(∇·$\vec{n}$) that is the curvature of the liquid–vapor interface, where $\vec{n}$ is the unit vector normal to the interface. In the interfaces, the volume tracking method was employed using the VOF algorithm [27]. The advection of the volume fraction was obtained from:

(3) $$\partial \psi /\partial t+\vec{v}\cdot \nabla \psi =0$$

The interfacial tension was calculated from the last term of the momentum equation [28]. The spatial derivatives were discretized by employing convective model [29]. The time step and maximum internal iteration were used as 1 μs and 30 per time step, respectively. A contact angle of 155° was adopted to reproduce the drop dynamics. A mesh resolution of the domain corresponded to at least 45 cells per drop diameter. The initial drop shapes with a constant volume were obtained from a formulation of the prolate spheroids and were patched in the computational domain.

Dimensionless parameters were used as follows: *x*, *y*, and *z* are the Cartesian coordinates normalized by drop radius, *R*. *a*, *b*, and *κ^−^*^1^ are the semiaxes and the radius of curvature of the *E*-surfaces, normalized by *R*, respectively. *w* is the drop’s width normalized by *D*, *τ_c_* is the residence time normalized by that of the spherical drop on flat surfaces, and *p* is the drop’s momentum in a certain axis, normalized by (*π*/6)*ρD*^3^*U* as:

*p_x_ = ∫ρ*[*v_x_* · sgn(*x*)]*ψ*_2_*dV* / [(*π*/6)*ρD*^3^*U*] (4)

where *v_x_* and sgn(*x*) are the velocity component and sign function of the axis respectively, *V* is the volume, *U* is the impact velocity, and *p_n_*, *p_t_*, and *p_z_* are the dimensionless momenta in the normal, tangential, and axial directions of the elliptic cylindrical coordinates, respectively.

To verify the numerical results, the experiment was performed using a nozzle-ring electrospray device. The setup for the ellipsoidal drop generation and impact on the solid surface was based on our previous works that studied the drop impact [21]. The inner shape of the ring was elliptic for the deformation of the drop by an electric field. To pinch the hanging drop off at the end of the nozzle (Hamilton 27 gauge), a pulse signal of 6–7 kV was applied to the electrodes for 7–10 ms, which produced the ellipsoidal drops. After being pinched off, the drop started to deform itself into the prolate spheroids that had the major axis of *x* when passing through the rings, as shown in Appendix A. The drop oscillated until the drop touched the solid surface. We fabricated superhydrophobic flat surfaces and circular wires with diameters of 2 mm, individually. The contact angles on the two surfaces were measured as 155 ± 3° and 155 ± 4°, respectively. The overall behaviors of the bouncing drops were captured using a high-speed camera (Fastcam SA3, Photron, Tokyo, Japan). The comparative outcomes from the simulation and experiment were obtained through the quantitative analysis of the temporally resolved widths of the horizontal axes. The bouncing dynamics of the drop obtained numerically were comparable to those obtained experimentally. Details of ellipsoidal drop generation and verification of the simulation are described in Appendix A.

Drop dynamics on the curved surfaces can be ruled by the inertio-capillary balance because the relevant dimensionless numbers give us Weber number *We* = *ρDU*^2^/*σ* = 14–34 and Ohnesorge number *Oh* = *μ*/(*ρDσ*)^1/2^ = 0.0027 ≪ 1. Therefore, the inertial and surface forces affect the drop dynamics, and the effect of the viscous force is negligible. The main parameters related to drop impact on curved surfaces range the drops’ ellipticity as *e* = 1 − *B*/*A* = 0~+0.53 for the case of the major axis parallel to the *z* axis, and *e* = −(1 − *B*/*A*) = −0.53~0 for the case of the major axis parallel to the *x* axis, where *A* and *B* are the major and minor axes of the ellipsoidal drops at the moment of impact, respectively. *E*-surfaces have the semiaxes of *a* = 2.0~4.0 and *b* = 0.4~16 in this study.

## 3. Results and Discussion

We established the model of *E*(*a*, *b*) surfaces that represent the semi-elliptic surfaces with the semiaxes of *a* and *b*, as shown in Figure 1c. The bouncing dynamics are highly affected by the geometric configurations between curved surfaces and drops. The ellipsoidal drops have the ellipticities of *e^+^* (*e* > 0) and *e^−^* (*e* < 0) when arranged to be the major axes of the *z* and *x*, respectively. Figure 1d indicates dimensionless surface curvatures that are derived from *κ*(*x*, *y*) = (*ab*)^4^/(*b*^4^*x*^2^ + *a*^4^*y*^2^)^3/2^, and the value at the apex is equal to an initial surface curvature *κ*_0_ = *b*/*a*^2^. Distinct from circular cylindrical surfaces with the constant *κ* (*a* = *b*), the curvatures of the *E*-surfaces are increasing (*a* > *b*) or decreasing (*b* > *a*) along the *x* axis. The color bar shown in Figure 1d means the magnitude of curvature, which will be used as the color of the *E*-surface later.

**Figure 2:**
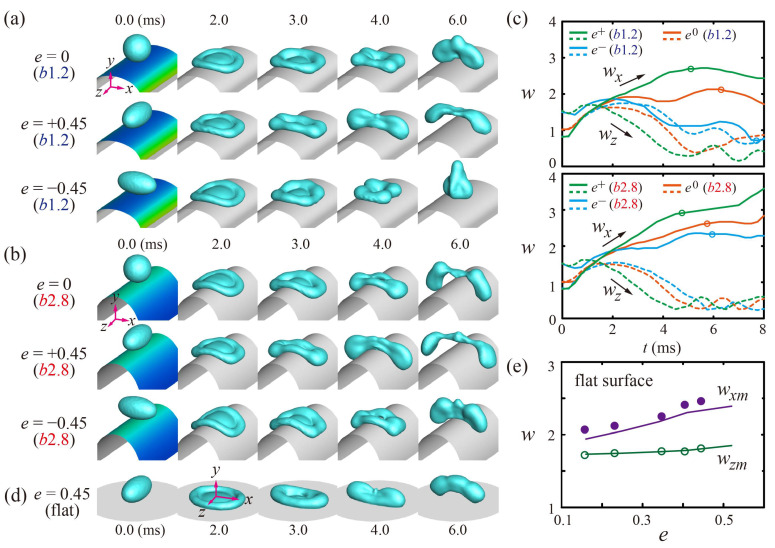
(**a**,**b**) Snapshots of the evolution of the drops on (**a**) *E*(2.0, 1.2) and (**b**) *E*(2.0, 2.8) surfaces at *We* = 24. (**c**) Temporal variations of the normalized widths, *w,* of the spherical (*e*^0^) and ellipsoidal drops with *e* = ±0.45 on the curved surfaces. The solid and dashed lines correspond to the *x*- and *z*-widths respectively, and open circles for each solid line denote the bouncing moments of the drops. (**d**) Ellipsoidal drops impacting on flat surfaces. (**e**) Maximum spreading widths (*w_m_*) on the flat surface as a function of *e*, obtained from the experiment (symbol) and simulation (line).

To understand the effects of the drops’ ellipticity and surface curvature on the bouncing behavior, we studied the temporal evolutions of the drops under *b* = 1.2 (*b*1.2) and 2.8 (*b*2.8), as shown in Figure 2a,b. *e^+^* drops spread wider along the *x* axis and leave the surface earlier than the spherical and *e^−^* drops on the surfaces. In addition, all the drops on the *b*2.8 surfaces exhibit further extensions along the *x* axis and bounce off the surface faster, compared with the drops on the *b*1.2 surfaces, as shown in Figure 2a,b at 6 ms. The bouncing characteristics are confirmed by the temporal variations of the *x* and *z* widths and the detachment times pointed out by using the single-circle symbols for each solid line, as shown in Figure 2c.

On the flat surfaces, as the limiting case (*κ*_0_ = 0), the *e^+^* (*e^−^*) drops have the hydrodynamic features of the spreading and retraction behaviors and the subsequent liquid alignment on the *x* axis (*z* axis) before bouncing, as shown in Figure 2d. The initially ellipsoidal shapes induce the predominant outward flow to the direction of the minor axis of the elliptical footprint during the impact. Thus, the *e^+^* drops stretch wider to the *x* axis than the *z* axis. In addition, the maximal extension of the *x* axis (*w_xm_*) is found later and greater than that of the *z* axis (*w_zm_*). Figure 2e represents the maximal extensions (*w_m_*) as a function of *e*, which shows that the drops have significant variations in *w_xm_*, but only slight variations in *w_zm_* in the simulation and experiment. The maximum relative error of the maximal extensions between numerical and experimental results is within 6%.

**Figure 3:**
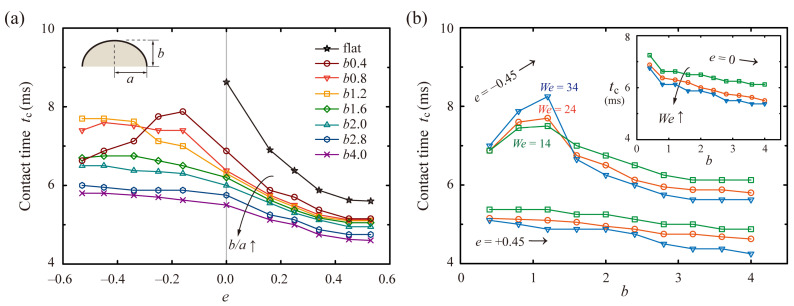
Effects of the surface anisotropy on the residence time. (**a**) *t_c_* on the flat and *a*2.0 surfaces under various *e* at *We* = 24. (**b**) *t_c_* of the drops with *e* = ±0.45 and spherical drops (inset) on *a*2.0 surfaces as a function of *b* under several *We*. The lines’ color and symbol correspond to *We* = 14 (green), 24 (red), and 34 (blue). The *e^−^* drops have peak values of *t_c_* around *b* = 1.2.

We investigated the effects of the surface anisotropy on the residence time of the drops. The *t_c_* on the flat and *a*2.0 surfaces (i.e., *E*(2.0, *b*) surface) are plotted in Figure 3a as a function of *e*, which reveals that rapid bouncing is found at high *b/a* and *e*. The *e^+^* drops provide an efficient pathway to reduce *t_c_* on *E*-surfaces, which presents a striking contrast to *e^−^* drops. On the *b*1.2 surfaces, for example, *t_c_* of the *e^+^* drops could reduce by approximately 19% and 40% below the spherical cases on the *b*1.2 surfaces and flat surfaces, respectively. On the *b*4.0 surfaces, *t_c_* of the *e^+^* drops could reduce by approximately 16% and 46% below the spherical cases on the *b*4.0 surfaces and flat surfaces, respectively. By contrast, *t_c_* of the *e^−^* drops generally increases with *|e|* on surfaces with increasing *κ* (*a > b*), whereas those are generally independent of *e* on surfaces with decreasing *κ* (*b > a*). For instance, *t_c_* of the *e^−^* drops increases up to 7.7 and 6.0 ms on the *b*1.2 and *b*2.8 surfaces, which correspond to increases of approximately 22% and 3% above the spherical cases on the same surfaces, respectively. Distinctively, *t_c_* of the *e^−^* drops on the *b*0.4 surfaces has a peak value at low *|e|.* This result is because the roles of the initial drop shape and surface curvature in symmetry-breaking in drop bouncing might be comparable to each other at the peak. 

Residence times on the curve surfaces result from a hydrodynamic interplay between the flow induced by effects of the drops’ initial shape and surface curvature. Assuming that *e^+^* drops are impacting on flat surfaces, the *e^+^* shapes induce a pronounced flow in the *x* axis, which could be intensified on the curved surfaces by the positive influence of the tangential momentum (*p_t_*) due to anisotropy of the surface. Conversely, assuming that *e^−^* drops are colliding on flat surfaces, the *e^−^* shapes induce the pronounced flow in the *z* axis, which could be suppressed on the curved surfaces by the negative influence of the *p_t_* that is orthogonal to the axial momentum (*p_z_*). Figure 3b shows *t_c_* of the *e^+^* and *e^−^* drops as a function of *b* under several *We*. As *We* increases, *t_c_* on the *b*1.2 surfaces increases up to 8.2 ms for *e^−^* drops and decreases down to 4.8 and 6.1 ms for *e^+^* and spherical drops, respectively. In addition, *t_c_* of the *e^−^* drops has peak values on the *b*1.2 surfaces at the given Weber numbers. As the height, *b,* increases, the role of the surface curvature in the asymmetric momentum transfer becomes dominant, thereby causing a monotonous decrease in *t_c_* of all the drops.

**Figure 4:**
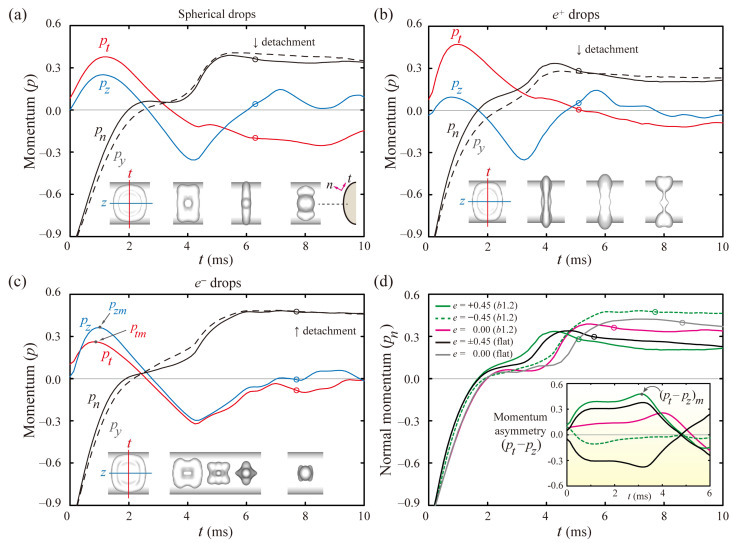
Axial momentum analysis for (**a**) spherical and (**b**,**c**) ellipsoidal drops with *e* = ±0.45 on *E*(2.0, 1.2) surfaces at *We* = 24. (**a**–**c**) Solid lines denote the dimensionless momenta in the normal (*p_n_*), tangential (*p_t_*), and *z* directions (*p_z_*) of the elliptic coordinates. Dashed lines correspond to the dimensionless *y*-momentum (*p_y_*). The insets indicate snapshots at distinct times. (**d**) Temporal evolutions of *p_n_* and the momentum asymmetry (*p_t_*–*p_z_* in the inset) indicate that *e^+^* drops show the rapid rising of *p_n_* and (*p_t_*–*p_z_*). Open circles for each solid line denote the bouncing moments of the drops.

To interpret the mechanism of reducing the residence time, the drop dynamics were analyzed in terms of the axial momenta. Figure 4 shows the dimensionless momenta in the normal to the surface (*p_n_*), tangential along the ridgeline (*p_t_*), and axial directions (*p_z_*) of the elliptic cylindrical coordinates, and *y*-directions (*p_y_*). The signs of *p* were imposed in the spreading (positive) and the retraction processes (negative) based on its definition. The insets represent snapshots at the distinct times. After touching the surfaces, the spherical drops enhance *p_t_* and *p_z_* and reach the maximum values, *p_tm_* and *p_zm_*, in the spreading process at nearly 1 ms, as shown in Figure 4a. Thereafter, the *p_t_* and *p_z_* become negative values at 3.5 and 2.6 ms, the onset times for the retraction process along their own directions, respectively. The gap between *p_t_* and *p_z_* could be closely relevant to the asymmetric mass and momentum transfer because a significant gap leads to the massive transfer in one direction and the subsequent liquid alignment with the same direction, as depicted in snapshots of Figure 4a at 6.0 ms. After this time, the drop leaves the surfaces at 6.3 ms, as pointed out by the single-circle symbol for each line.

*e^+^* drops expand the discrepancy between *p_t_* and *p_z_* and complete the liquid alignment, as shown in Figure 4b, at 4.0 ms. The drops enhance the asymmetries of the mass and momentum, thereby leading to fast bouncing at 5.1 ms, which is in marked contrast to *e^−^* drops. This is because the *e^−^* drops display a minor difference between *p_t_* and *p_z_*, even until nearly 7.0 ms, and are thereby detached from the surfaces at 7.7 ms, as presented in Figure 4c. The switching times of the *p_t_* and *p_z_* required to change from the spreading to retraction process are comparable to each other for the *e^−^* drops. The snapshots of the inset at 4–8 ms indicate that the retraction dynamics are approximately axisymmetric, comparable to the features of the spherical drops.

Figure 4d shows the temporal evolutions of *p_n_* and momentum asymmetry (*p_t_*–*p_z_*, in the inset) on the flat and *E*-surfaces, which reveals that the *e^+^* drops show the rapid rising of *p_n_* and (*p_t_*–*p_z_*) at the earliest time and detach from the surfaces at the lowest *t_c_* among the several drops. The momentum asymmetry of the drops shown in the inset of Figure 4d is also closely connected to the bounce speed. The enhanced asymmetry of the momenta is found at high values of |*p_t_*–*p_z_*|. Asymmetry of the spherical and *e^+^* drops reach the maximum values of (*p_t_*–*p_z_*)*_m_*, as indicated by the arrow of the inset. Meanwhile, the maximal asymmetry of the *e^−^* drops on the *b*1.2 (increasing *κ*) surfaces occurs during the spreading because of the dominant role of the drops’ initial shape in the hydrodynamics at the low surface curvatures. We suggest that the momentum asymmetry can be convincing evidence for the decrease in the residence time. Details in the velocity fields for ellipsoidal drops are described in Figure S1 of the Supplementary Materials.

**Figure 5:**
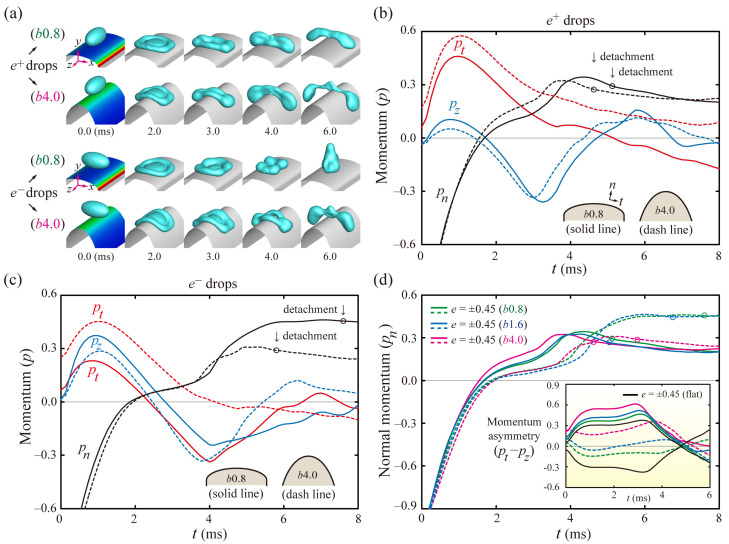
Effects of the height, *b*, of the curved surface on the residence time. (**a**) Shape evolutions of the drops with *e* = ±0.45 on *E*(2.0, 0.8) and *E*(2.0, 4.0) surfaces at *We* = 24. (**b**,**c**) Axial momenta of the drops with (**b**) *e* = +0.45 and (**c**) *e* = −0.45 on the *b*0.8 (solid line) and *b*4.0 surfaces (dashed line). The illustrations of the insets indicate the surface outlines. (**d**) Temporal variations of *p_n_* and the momentum asymmetry (*p_t–_p_z_*, in the inset) for *e^+^* (solid line) and *e^−^* (dashed line) drops on *a*2.0 surfaces. Single-circle symbols for the *p_n_*-curves denote the bouncing moments of the drops.

To better elucidate the effects of the height, *b,* on the residence times, we examined the evolutions of the shapes on *b*0.8 and *b*4.0 surfaces. Figure 5a shows that *e^+^* drops on the *b*4.0 surfaces exhibit severe elongation to the *x* axis, compared with those on the *b*0.8 surfaces, as shown at 6 ms. On the contrary, *e^−^* drops on the *b*0.8 surfaces behave as roughly axisymmetric by evolving into the vertical liquid column before bouncing, whereas those on the *b*4.0 surfaces massively redistribute to the *x* axis and then depart from the surfaces early. Figure 5b,c indicate the temporal variation of the axial momenta of the ellipsoidal drops on *b*0.8 (solid line) and *b*4.0 surfaces (dashed line). Figure 5d shows the temporal variations of *p_n_* and (*p_t_*–*p_z_*) for several drops. The solid and dashed lines correspond to the momenta of the *e^+^* and *e^−^* drops, respectively. The discrepancy between *p_t_* and *p_z_* of the *e^+^* drops on the *b*4.0 surface is more intensified than that on the *b*0.8 surfaces. Meanwhile, the discrepancy of the *e^−^* drops on the *b*0.8 surfaces has negative signs because the role of the drops’ initial shape is dominant in the hydrodynamics at the low surface curvatures, as discussed earlier. The *e^+^* drops on the *b*4.0 surfaces show the fastest growths of *p_n_* and (*p_t_*–*p_z_*) among several drops. As the height, *b,* increases, the initial surface curvature and the momentum asymmetry increase. 

**Figure 6:**
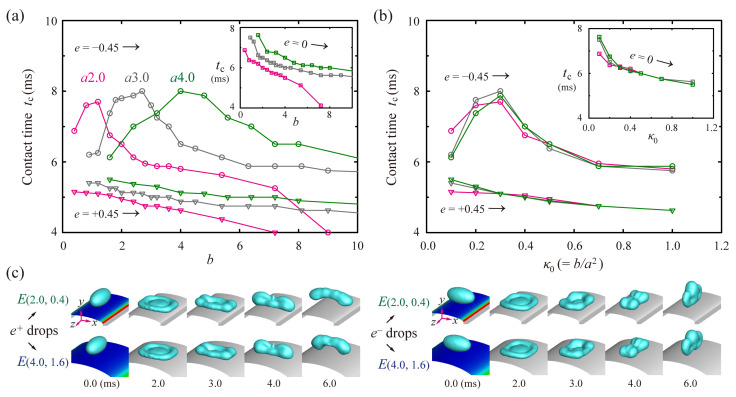
Dependency of residence time on the initial surface curvature, *κ*_0_. (**a**) *t_c_* of the drops with *e* = ±0.45 and spherical drops (inlet) as a function of *b* under *a* = 2.0 (pink), 3.0 (gray), and 4.0 (green). (**b**) Residence times of the latter drops as a function of *κ*_0_. The *t_c_* is roughly gathered around one line for each drop when plotted with *κ*_0_. (**c**) Snapshots of the drops with *e* = ±0.45 on *E*(2.0, 0.4) and *E*(4.0, 1.6) surfaces with *κ*_0_ = 0.1 at *We* = 24.

Figure 6a shows *t_c_* of the ellipsoidal drops with *e* = ±0.45 and spherical ones (insets) under three different *a*. The *t_c_* of the *e^−^* drops has peaks around *b* = 1.2, 2.7, and 4.0 at *a* = 2.0, 3.0, and 4.0, respectively. When *t_c_* is plotted by the initial surface curvature, *κ*_0_, the *t_c_* is roughly gathered around one line for each drop, as shown in Figure 6b. Thus, residence times could be characterized in terms of *κ*_0_. The figure reveals that the drops have the only slight deviation of *t_c_* between the surfaces with the different widths at high *κ*_0_, in spite of the relatively high deviation of *t_c_* at low *κ*_0_. Figure 6c shows shape evolutions of the drops on *E*(2.0, 0.4) and *E*(4.0, 1.6) surfaces, commonly with *κ*_0_ = 0.1. The figure indicates that the ellipsoidal drop dynamics on the two surfaces are similar to each other. Accordingly, the conditions of the high *κ*_0_ and high *e* play an important role in the symmetry-breaking in the mass and momentum distribution. Temporal variations of the momentum symmetries on the several *E*-surfaces with *κ*_0_ = 0.1, including *E*(10, 10) surfaces, were comparable to each other, which is described in Figure S2 of the Supplementary Materials.

**Figure 7:**
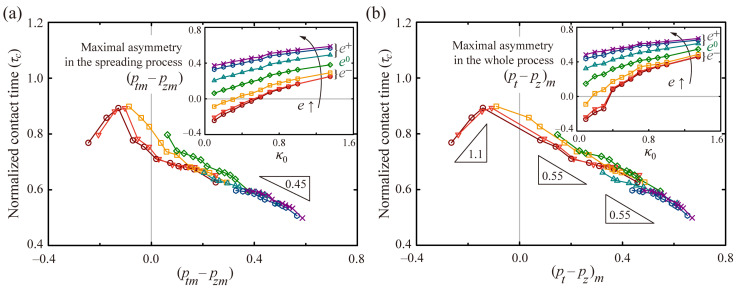
Normalized residence times, *τ_c_*, as a function of the maximal asymmetry of the momenta for varying *b* at *a* = 2.0 and *We* = 24. The symbols and lines represent the several ellipticities following: *e* = −0.53 (brown), −0.45 (red), −0.25 (orange), 0 (green), +0.25 (cyan), +0.45 (blue), and +0.53 (purple). (**a**) Normalized residence times as a function of (*p_tm_*–*p_zm_*) that is the difference between the maximum momenta in the spreading process. (**b**) Normalized residence times as a function of (*p_t_*–*p_z_*)*_m_* that is the maximum difference between the momenta in the whole spreading and retraction processes. The insets in (**a**,**b**) indicate the maximal asymmetries as a function of *κ*_0_.

The residence time can be closely related to the maximal asymmetry of the momenta. We plotted the normalized residence time, *τ_c_*, with *P_sp_*, where *P_sp_* = (*p_tm_*–*p_zm_*), the difference between the maximum momenta within the spreading process, as shown in Figure 7a. The solid lines and symbols represent the drops’ several ellipticities ranging from −0.53 to +0.53. The inset represents the plot of *P_sp_* as a function of *κ*_0_, which reveals that *P_sp_* has a significant value at high *e* and high *κ*_0_. Additionally, the *τ_c_* is generally in inverse proportion to *P_sp_*, partly satisfying the slope of −0.45 at very high *P_sp_*. However, when the *τ_c_* is plotted by *P_wh_*, it is well-fitted by the single lines with slopes of −0.55 and 1.1 at the positive and negative *P_wh_* respectively, as shown in Figure 7b, where *P_wh_* = (*p_t_*–*p_z_*)*_m_*, the maximum difference between the momenta within the whole spreading and retraction processes. The spherical and *e^+^* drops hold the slope of −0.55. Meanwhile, the *e^−^* drops hold the slope of 1.1, retaining the negative *P_wh_* at low *κ*_0_, as shown in the inset of Figure 7b. Therefore, the maximal asymmetry, *P_wh_*, can serve to determine the reduction in *τ_c_* by emphasizing the richness of the physics of how surface curvatures and initial drop shape affect the bouncing features. We also concluded that the drops’ ellipticity is considered capable of controlling the drop mobility by offering a broader range of the *τ_c_* (approximately from 0.5 to 0.9), compared with the spherical case (*τ_c_* ≈ 0.6–0.8).

As an additional note, we found the shape evolution of the oblate drops on curved surfaces to compare with those of the prolate spheroidal shapes, which showed that the dynamics of the prolate and oblate drops were similar. In addition, we also predicted the bouncing dynamics of ellipsoidal drops that were rotated 45° on the *y* axis, and indicated that the symmetry-breaking bouncing on *E*-surfaces was also significantly affected by anisotropic curvatures. Details of the impact dynamics were described in Figures S3 and S4 of the Supplementary Materials.

## 4. Conclusions

We investigated the bouncing features and *t_c_* of ellipsoidal drops on the *E*-surfaces, compared with spherical drops. The numerical results revealed that the dynamics highly depended on the geometric configuration between the elliptical curves and ellipsoidal drops. The *t_c_* obtained from the different geometric relations could be determined by the hydrodynamic interplay between the influence of the initial drop shape and surface curvature. To better understand the roles of the two factors in drop dynamics, we investigated the evolutions of shapes and momentum asymmetries of the drops on various *E*-surfaces, which revealed that the rapid bouncing appeared at high *κ*_0_ and high *e*. *t_c_* of *e^+^* drops on *E*(2.0, 4.0) surfaces could diminish by approximately 46% below the spherical cases on flat surfaces at *We* = 24, which presented the enhancement of asymmetries of the mass and momentum. *t_c_* of *e^−^* drops generally increased with *|e|* on surfaces with increasing *κ*, whereas those were independent of *e* on surfaces with decreasing *κ*, in general. In addition, we found that the residence time could be closely related to the maximal asymmetry of the momenta, which showed that *τ_c_* decreased roughly linearly against (*p_t_*–*p_z_*)*_m_* for the spherical and *e^+^* drops. The drops’ ellipticity could have the ability to adjust the repellency from surfaces by covering a broad range of the residence time beyond the spherical cases. We believe that reshaping of the ellipsoidal drops will provide new insight into the strategies for further lower retention on bioinspired surfaces, such as an array of cylinders or corrugated surfaces. The fundamental understanding of the drop dynamics will be able to assist practical applications, such as dropwise condensation, anti-corrosion, and anti-icing.

## Figures and Tables

**Figure A1:**
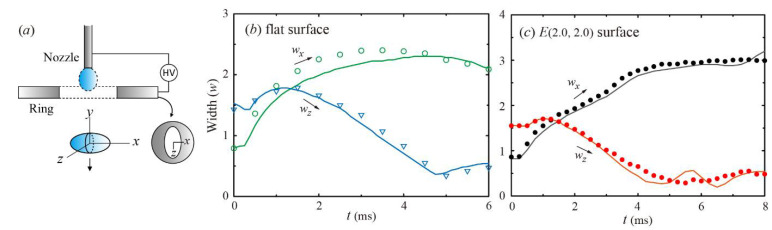
Verification of numerical results. (**a**) Illustrations of the ellipsoidal drop generation. (**b**) Spreading and retraction dynamics of the drops with *e* = 0.45 on the flat surfaces at *We* = 24. (**c**) Spreading and retraction dynamics of drops with *e* = +0.45 on cylindrical surfaces with *a = b =* 2.0. The *w* represents the width of the *x* axis (*w_x_*) and *z* axis (*w_z_*), normalized by drop diameter. The results are obtained from the experiment (symbol) and simulation (line).

## Supplementary Materials

The following are available online at https://www.mdpi.com/article/10.3390/polym13172940/s1, Figure S1: velocity fields of ellipsoidal drops, Figure S2: momentum asymmetry on several surfaces at the constant *κ*_0_, Figure S3: shape evolutions of oblate ellipsoidal drops on *E*-surfaces, Figure S4: shape evolutions of 45°-rotated ellipsoidal drops on *E*-surfaces.

## Appendix A

To verify the numerical results, the experiment was performed using a nozzle-ring electrospray device. When a pulse signal of high voltage was applied between the electrodes, the hanging drop from the nozzle began to stretch out toward the ring. After being pinched off, the drop started to deform itself into the prolate spheroids that had the major axis of *x* when passing through the rings, as shown in Figure A1a. The bouncing dynamics of the drop obtained numerically were similar to those obtained experimentally, as shown in Figure A1b,c.
