# Supplementary material for: Symmetry-Breaking Drop Bouncing on Superhydrophobic Surfaces with Continuously Changing Curvatures
Source: Polymers (Basel). 2021 Aug 31;13(17):2940. doi: 10.3390/polym13172940 (PMC8434175; doi:10.3390/polym13172940)
Supplement: Supplementary file 1 [file polymers-13-02940-s001.zip › polymers-1362165-SI.pdf]

Supplementary Materials for
"Symmetry-Breaking Drop Bouncing on Superhydrophobic Surfaces with Continuously Changing Curvatures"

WooSeok Choi and Sungchan Yun

Department of Mechanical Engineering, Korea National University of Transportation, Chungju 27469, Republic of Korea

S2: Figure S1. Velocity field distributions of e^{+} and e^{-} drops.

S3: Figure S2. Momentum asymmetry on several surfaces at the constant κ_0.

S4: Figure S3. Shape evolutions of the oblate ellipsoidal drops on E-surfaces.

S5: Figure S4. Shape evolutions of 45°-rotated ellipsoidal drops on E-surfaces.

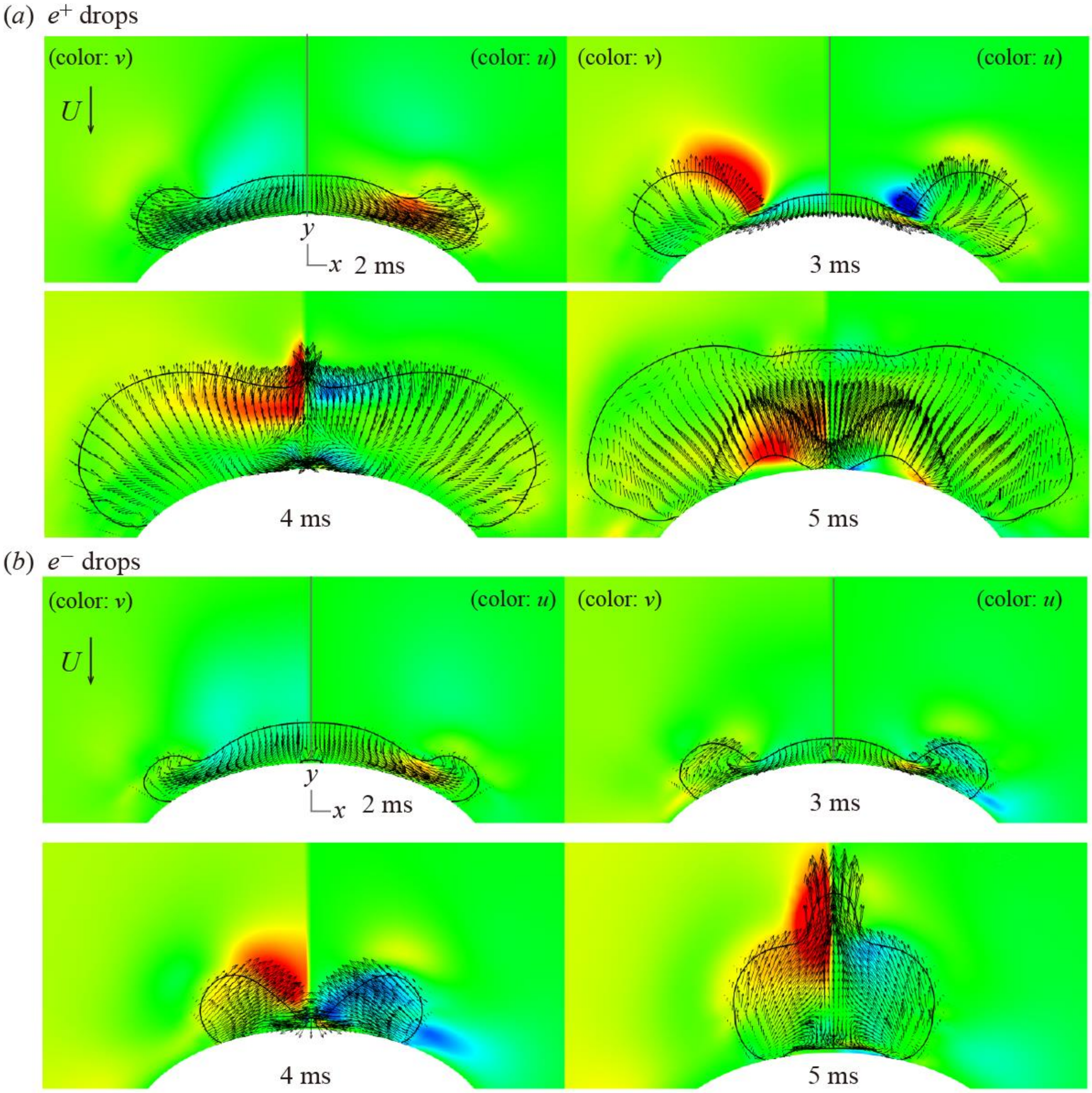

Figure S1. Velocity field distributions (arrow) and the magnitude of the velocity (color) of the drops with (a) $e = +0.45$ and (b) $e = –0.45$ on $E(2.0, 1.2)$ surfaces at $We = 24$. U, u, and v correspond to the initial impact velocity, x-velocity, and y-velocity, respectively. The left and right columns of the contours represent the v and u, respectively. The intensity of the color is clipped at the initial impact velocity $\pm U$ (red: high; blue: low).

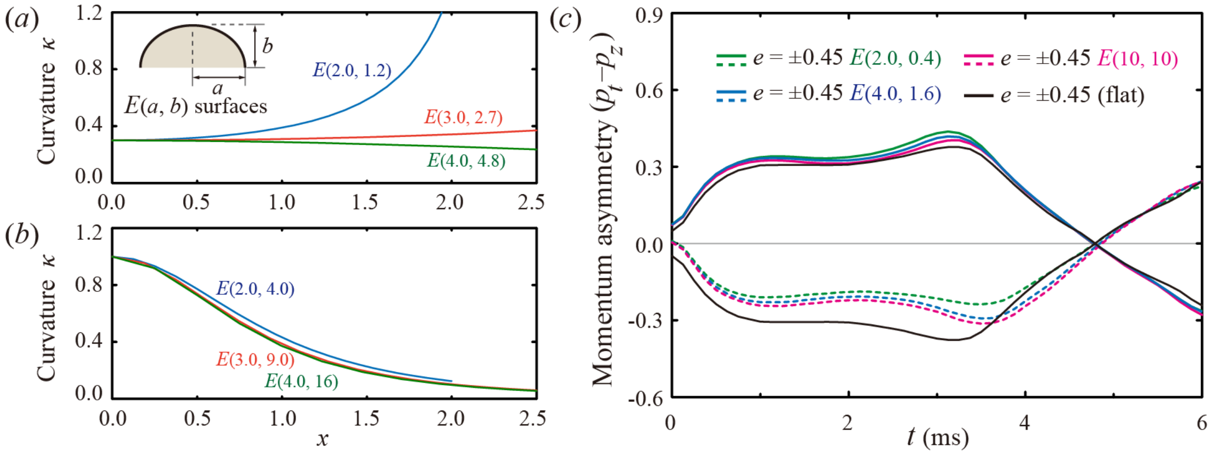

Figure S2. Dimensionless curvature κ along the x axis at the initial surface curvature of (a) $\kappa_0 = 0.3$ and (b) $\kappa_0 = 1.0$. The high deviation of κ between the surfaces at the low κ_0, whereas the slight deviation of κ between the surfaces at the high κ_0. (c) Temporal variations of the momentum asymmetry ($p_t - p_z$) at e = +0.45 (solid line) and e = –0.45 (dashed line) on the surfaces at κ_0 = 0.1. The momentum asymmetries on several E-surfaces, including the E(10, 10) surfaces, are comparable to each other.

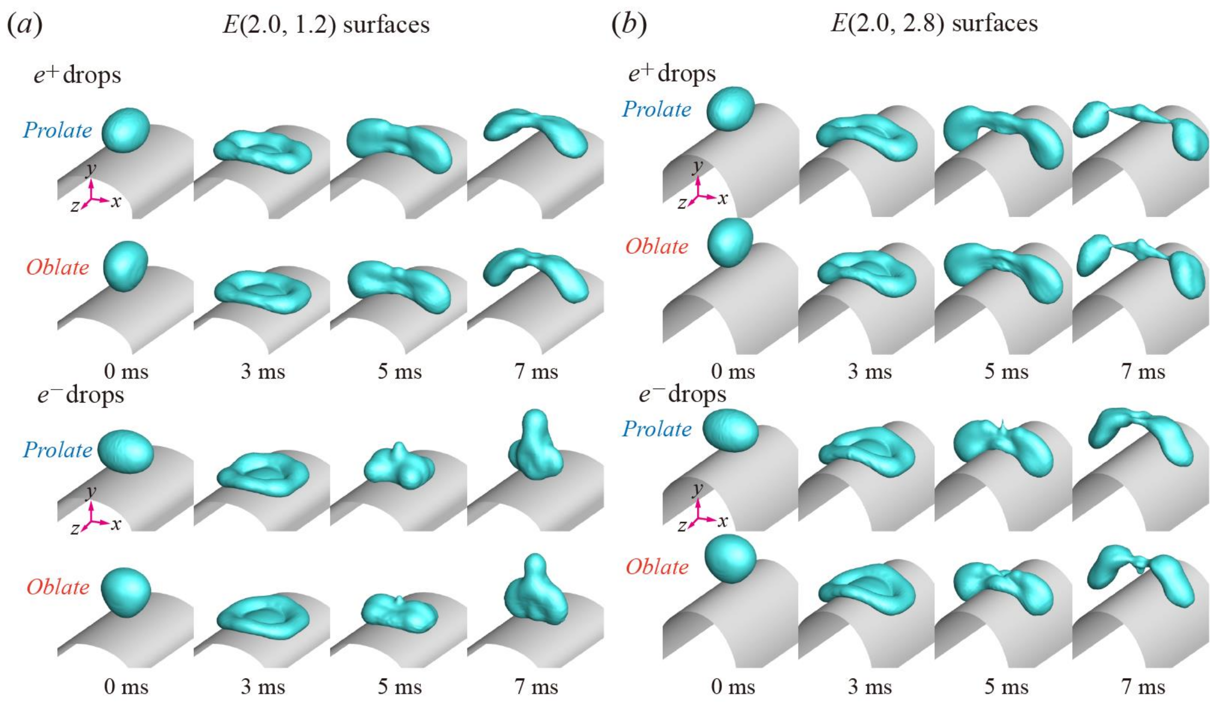

Figure S3. Bouncing dynamics on (a) *E*(2.0, 1.2) and (b) *E*(2.0, 2.8) surfaces for prolate and oblate spheroidal drops with $e = \pm 0.25$ at $We = 24$. The shape evolution and residence time of the prolate drops are comparable to those of the oblate drops.

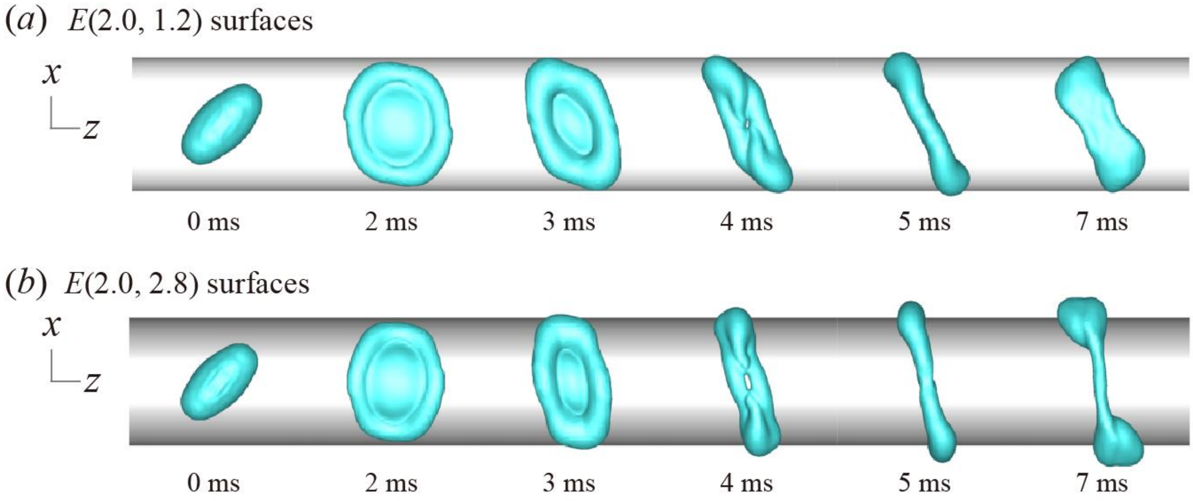

Figure S4. Snapshots of the bouncing dynamics of 45°-rotated prolate drops around the *y* axis on (a) *E*(2.0, 1.2) and (b) *E*(2.0, 2.8) surfaces at *We* = 24. The drops complete the liquid alignment along the direction biased slightly to the *x* axis because of a pronounced flow to the axis induced by the curvature, as shown at 5 ms. The drops leave the *b*1.2 and *b*2.8 surfaces at 5.3 and 5.2 ms, respectively.
